# A qualitative study to evaluate the preparedness of community paediatricians for genomic medicine in England - ready for take-off?

**DOI:** 10.1007/s12687-025-00781-8

**Published:** 2025-03-12

**Authors:** Sophie Marlowe, Melissa Hill, Michelle Peter, Celine Lewis

**Affiliations:** 1 North East Thames Clinical Genetics Service, Great Ormond Street Hospital for Children NHS Foundation Trust, London, UK; 2 North Thames Genomic Laboratory Hub, Great Ormond Street Hospital for Children NHS Foundation Trust, London, UK; 3 Genetics and Genomic Medicine, UCL Great Ormond Street Institute of Child Health, London, UK; 4 Population, Policy and Practice Department, UCL Great Ormond Street Institute of Child Health, London, UK

**Keywords:** Genomic medicine, Non-genetics specialists, Whole genome sequencing, Community paediatrics

## Abstract

Genomic medicine (GM) was mainstreamed across the National Health Service (NHS) in England in 2018. Non-genetics healthcare professionals can now incorporate genomic testing including whole genome sequencing (WGS) into their clinical practice. This study was conducted to evaluate the preparedness of community paediatricians (CPs) for GM. Semi-structured interviews, using a topic guide informed by the Consolidated Framework for Implementation Research, were conducted with 17 CPs working in the NHS to explore issues related to preparedness and confidence. Data were analysed using thematic template analysis. The codebook included both inductive and deductive codes informed by the Capability, Opportunity and Motivation Behaviour model (COM-B), an implementation theory to explain behaviour change. The majority of participants perceived a net benefit from GM in terms of improving clinical management and information provision for patients and families and were receptive to using GM in their clinical practice. However, there was wide variation across trusts in CP preparedness for genomic medicine for reasons including lack of time and resources, notably workforce support. Many also lacked confidence in the skills required to deliver GM, and did not see GM as a priority. Most participants felt that they had access to GM education, but the main challenge was finding the time to engage with it. Strategies related to fiscal measures, enablement, training and education could help to address these early obstacles. Our findings may be relevant to clinicians in other non-genetic specialties integrating GM into their clinical practice not only in the UK NHS but more globally.

**Supplementary Information:**

The online version contains supplementary material available at 10.1007/s12687-025-00781-8.

## Introduction

**Fig. 1:**
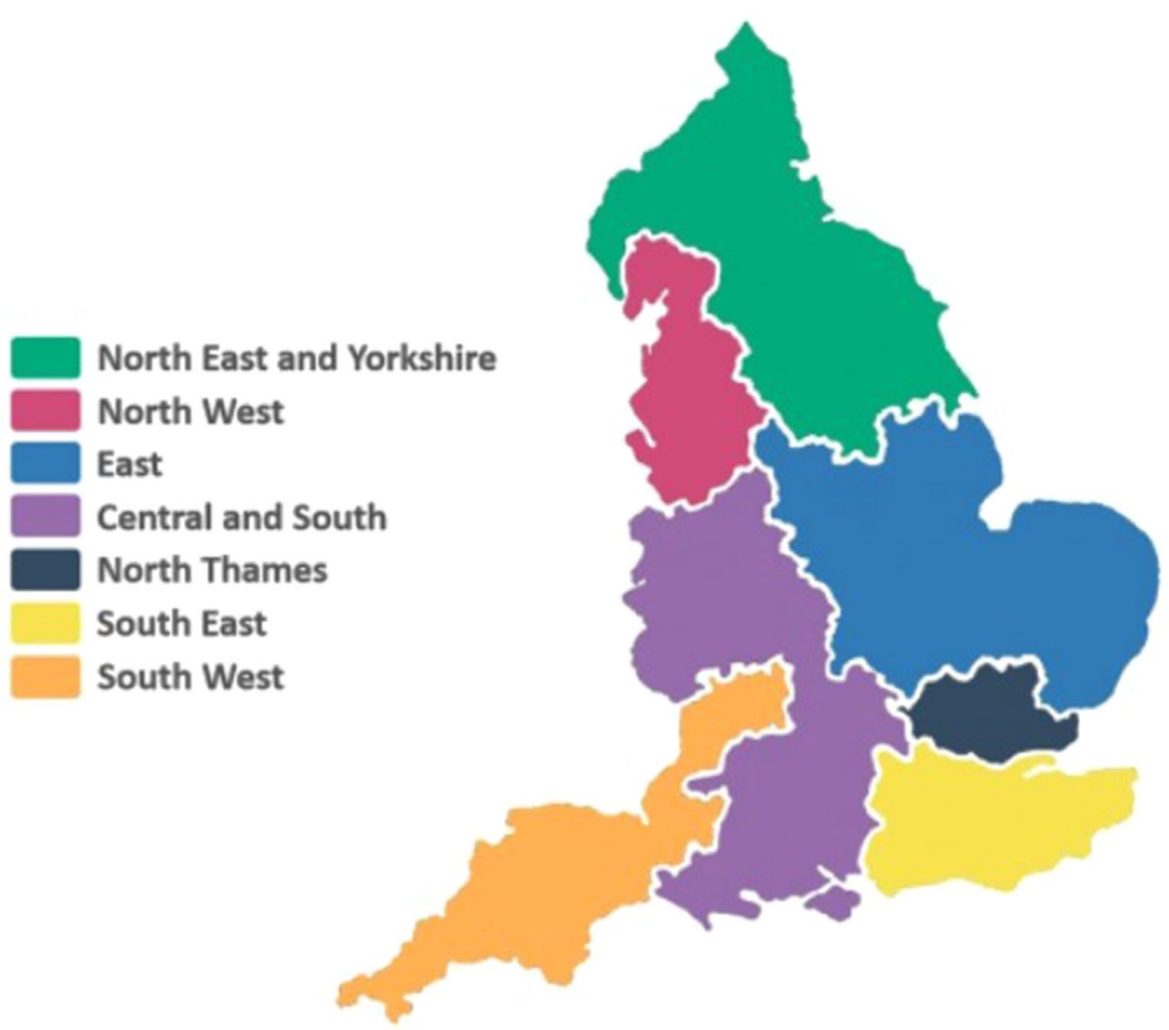
Image depicting the geographical spread of the GMSAs (NHS North West Genomic Medicine Service Alliance, 2025)

The Genomic Medicine Service (GMS) was launched in in 2018, to create a step change in the use of genomic technologies in the National Health Service (NHS), including offering high throughput whole genome sequencing (WGS) as part of routine care for both rare disease and cancer. The aim of the GMS was to create a national genomic testing service through established clinical and laboratory networks, providing equitable and personalised care for patients regardless of their geographical location within England (Hill 2018; NHS England 2022b). Genetic services in England were reconfigured and a new system was implemented, which included the introduction of seven Genomic Laboratory Hubs (GLHs) and Genomic Medicine Service Alliances (GMSAs) (Fig. 1), that are each responsible for the genomic testing within a given geographical region (NHS England 2022b). Each GLH/ GMSA serves multiple hospital trusts (a legal entity that provides healthcare services to the National Health Service (NHS) in England and Wales) and clinical genetics departments within the region who coordinate testing; thus centralising and aiming to standardise testing nationally (NHS England 2022b). Tests offered within the new service include single gene tests, gene panels, whole genome microarray, and wider whole exome (WES) and whole genome sequencing (WGS).

Mainstreaming genetic and genomic testing is a key part of the new service, whereby non-genetics specialists are able to order diagnostic genomic tests for their patients using the National Genomic Test Directory (NHS England 2022a). This might include, for example, paediatricians ordering an epilepsy gene panel for children who have seizures suspected to have a monogenic cause. The rationale for this is that genomic medicine (GM) will be more integrated into routine clinical care, and genomic testing will become more streamlined and accessible for patients. Mainstreaming of genetics into routine clinical care requires that non-genetics specialists receive adequate genetics education and training to feel competent and confident in providing these services to patients (Davies 2016). Typically, UK-trained clinical doctors will have touched on some genetics theory in their undergraduate degrees, but the level and content differ between institution. There is also likely to be a disparity in the length of time since their study, dependent on their current career stage. Each non-genetic specialist’s level of exposure to genetics in their day-to-day role also differs widely. This disparity in genetics education and experience amongst NHS HCPs is likely to have an impact on how services are delivered within the new GMS.

Community paediatricians (CPs) are expected to be key HCPs involved in genomics mainstreaming. As specialist children’s doctors working across different hospital trust locations and health centres, they assess and manage children with complex health needs, including children with neurodevelopmental conditions, such as epilepsy and intellectual disability, and children diagnosed clinically with multiple medical conditions that are part of a syndrome. CPs also see children for whom a diagnosis is being investigated, but unable to be made. CPs therefore have the potential for increased involvement in mainstreaming for diagnosis of these complex health conditions. As implementation of GM evolves, CPs will have a growing active involvement with patients for whom WES and WGS could be indicated.

To help guide non-genetics HCPs through the implementation of GM in their practice, user guides and educational resources are available online; this includes higher level information about WGS and documents for use, such as record of discussion forms and test order forms (Genomics Education Programme 2022; Genomics England 2025).

Additionally, the GMSAs have an educational role and support mainstreaming HCPs within their assigned geographical region. This may include organising training sessions, including webinars, ‘lunch-and-learn’ sessions, and educational drop-in sessions (South East Genomics 2022; Virtual course archives; NHS England 2024).

Studies on the preparedness of non-genetics HCPs have been conducted in countries including Australia (Nisselle et al. 2021; Jayasinghe et al. 2020; McClaren et al. 2020a, b; Crellin et al. 2019), Canada (Carroll et al. 2016, 2019; Chow-White et al. 2017; Mainous et al. 2013), the US (Douma et al. 2016; Johnson et al. 2017; Scheuner et al. 2022; Chambers et al. 2015; Mainous et al. 2013; Christensen et al. 2016^)^, the Netherlands (Nippert et al. 2011; Bokkers et al. 2022), Germany (Nippert et al. 2011), France (Nippert et al. 2011), Sweden (Nippert et al. 2011) and the UK (Nippert et al. 2011; Bakir et al. 2019; Mwale and Farsides 2021). The findings highlight varying knowledge levels about GM amongst a range of non-genetics HCPs, although specific groups of HCPs feel more prepared to use it in their practice than others. Many non-genetics HCPs foresee clinical benefits of GM for their patients but do not feel confident in knowing how to implement it into their roles. They suggest more educational opportunities and better guidelines to improve their knowledge and preparedness for engaging with GM.

A number of theories, models and frameworks in implementation research have been developed in recent years to describe translating research into practice, explain what influences implementation outcomes, and to design and implement targeted interventions (such as educational opportunities and improved guidelines to improve preparedness) (Nilsen 2015). New research is also needed to consider the confidence, competence and preparedness of non-genetics HCPs following the implementation of the NHS GMS (Bokkers et al. 2022). Importantly, there have been no studies conducted exclusively with CPs, a key part of the mainstreaming workforce, providing a timely opportunity for this research. Using implementation theories, models and/or frameworks to understand the experience of CPs tasked with delivering genomic medicine is therefore worthwhile.

This study is part of a wider mixed methods evaluation of the NHS GMS for paediatric rare diseases (Lewis et al. 2022). This article reports findings from an interview study with NHS-based CPs. The primary aim of this study was to evaluate the preparedness and confidence of CPs for GM by identifying their current attitudes towards GM, their experience of offering genomic testing in their practice, and consider ways to improve the preparedness and confidence of CPs for GM.

## Methods

### Ethical approval

The study was registered with UCL Great Ormond Street Institute of Child Health Research and Development as a Service Evaluation Study (21PP11). The study was performed in accordance with the ethical standards as laid down in the 1964 Declaration of Helsinki and its later amendments.

### Study design

This was a qualitative interview study using purposive sampling to recruit CPs working in the NHS; this sampling method was appropriate to facilitate the recruitment of participants with the required characteristics (i.e., CPs working in the NHS across a range of geographical locations). Semi-structured one-to-one interviews were chosen to facilitate an in-depth exploration of healthcare professionals’ preparedness for genomic medicine.

### Study setting

All participants were working within the NHS in England.

### Theoretical approach

Data collection and analysis were guided by two theoretical approaches used in implementation science We used a determinant framework (the CFIR) to identify the types of determinants (system and individual) which act as barriers and enablers to implementing a service, and an implementation theory (the COM-B) to provide understanding and/or explanation of aspects of implementation (Nilsen 2015).

#### The consolidated framework for implementation research (CFIR)

The CFIR was used to devise the interview topic guide (see Supplementary Material) for the interviews and is an evidence-based determinant framework used in implementation design and evaluation of an innovation (Damschroder et al. 2009). This tool was chosen to ensure that the topic guide questions allowed us to systematically assess the factors that influence implementation of GM amongst CPs. It is also being used to guide data collection and analysis in a number of other sub-studies within this programme of work and would therefore allow for comparison across studies (Lewis et al. 2022). It has been used in other studies looking at the adoption of genomics. For example, Zebrowski et al. used the CFIR in a qualitative study to identify barriers and facilitators related to system-level factors that have played a role in the implementation of genomic medicine in the United States of America (Zebrowski et al. 2018). And Jayasinghe et al. used the CFIR to guide the questions included in a survey disseminated to nephrologists to determine their preparedness in implementing genomics (Jayasinghe et al. 2020, Attitudes and Practices of Australian Nephrologists Toward Implementation of Clinical Genomics). The tool has five domains, comprising different constructs that can be applied to the implementation of an innovation. The domains most pertinent to this study were “Inner Settings” and “Characteristics of Individuals” which look broadly at the organisation in which participants are embedded, but also their individual characteristics and attitudes towards the implementation.

#### The COM-B behaviour change wheel

The capability, opportunity, motivation behaviour (COM-B) behaviour change wheel was chosen to guide the development of the codebook used for data analysis. It was chosen as it focuses on individual attitudes and behaviours that may or may not lead to behaviour change. The COM-B is a model for defining the core conditions (‘specific sets of actions’) that influence behaviour change, as developed by Michie et al. (Michie et al. 2011). The three main interacting conditions capability, motivation and opportunity, collectively determine whether a behaviour, such as the integration of genomic medicine into practice, occurs. The behaviour change wheel builds on this by providing a structured framework that links these conditions to specific intervention functions (e.g. education, training, enablement) and policy categories (e.g. guidelines, service provision, regulation) deigned to address gaps in these areas. In this way, the COM-B model helps diagnose where preparedness gaps exist, which the behaviour change wheel guides the selection of evidence-base strategies to target these gaps.

This model has previously been applied to different aspects of behaviour change in healthcare (Surr et al. 2020; McDonagh et al. 2018) and has been identified as a useful tool for designing educational strategies around the preparedness of HCPs for GM (Samuel et al. 2022). McClaren et al. used the COM-B to inform the development of a national survey assessing physician preparedness for GM in Australia and their preferences for continuing education (McClaren et al. 2020b).

### Recruitment

Prospective participants were sent study invitations through mailing lists for NHS email networks and professional bodies known to have links to the study population, who were all paediatricians, including community paediatricians, all working in the NHS where exposure to the genetic and genomic testing through the GMS is likely, but levels of experience and engagement vary. These networks included the North Thames GMSA, the Royal College of Paediatrics and Child Health, and the British Academy of Childhood Disability. A study advert was also included in eBulletins and newsletters for these professional bodies. An agnostic approach to recruitment was taken because a range of viewpoints from CPs across England was desired. We wanted the recruitment process to be far-reaching to any eligible CP who was interested. However, unfortunately we did not receive any responses via these methods. We therefore initially targeted community paediatricians known to one of the authors and one of the project advisory team members. A snowball approach was then used to maximise recruitment through encouraging participants to suggest colleagues who might be interested. In total, 23 potential participants were contacted by either SM or CL via email and sent a participant information sheet. All those that agreed to participate either provided written consent or consent was verbal and recorded on a digital audio-recorder.

### Data collection

Semi-structed interviews were carried out by SM and CL over videoconferencing software or by telephone. Interviews were digitally recorded, transcribed, and checked before the coding process began. The topic guide covered participants’ general knowledge about the GMS, including: its aims, experience with GM, involvement with WGS, attitudes towards WGS in community paediatrics, confidence in their competence around GM, and how supported for GM they feel their department is. We also asked participants to self-rate their experience of genomics with the response options of ‘none’, ‘some’ and ‘a lot’.

### Data analysis

Data were analysed using thematic template analysis (Brooks et al. 2015), using a coding template formed using the original COM-B domains. Template analysis is a flexible yet structured method for thematic analysis that involves developing a coding ‘template’ to organise and interpret qualitative data. It allows for the integration of both deductive and inductive coding approaches, making it ideal for studies guided by pre-existing theoretical models like COM-B. It was important to allow for inductive codes to be captured which might not have been captured if using the COM-B domains alone. A coding reliability approach was used with the codebook and applied consistently to code all the data (Braun and Clarke 2019).

The first draft of the codebook included the six COM-B sub-themes, along with descriptions of how each sub-theme was defined in the particular context of genomic testing (see Findings section for definition). We mapped the constructs preparedness and confidence – two of the key focus areas of the study - onto the COM-B model by conceptualising preparedness as reflecting an individual’s overall readiness to engage in a behaviour (encompassing aspects of capability), and confidence as aligning with the motivation component of the COM-B model, particularly within the domain of reflective motivation which includes self-efficacy and beliefs about capabilities. Within the COM-B six sub-themes, we developed codes inductively from the data which were more granular than their over-arching sub-theme. This combination approach (using both deductive and inductive coding) encouraged generation of new ideas and exploration of novel areas where there is a lack of existing knowledge available (Noble and Mitchell 2016; Surr et al. 2020).

The first iteration of the codebook was developed by SM and then applied to three transcripts by SM and CL. This led to a second iteration of the codebook which was then discussed between SM, CL, MH and MP. SM then coded the remainder of the transcripts, and CL double-coded a proportion of the transcripts to ensure consistency of application to the codebook (Braun and Clarke 2019).

Coding was manually done using Microsoft Word, and transcripts were then re-coded using Nvivo 12 Pro. The themes were reviewed to ensure data comparability, at which point some inductive codes were merged. The results are presented according to the final ‘template’.

## Results

### Participant characteristics

In total, 17/23 participants, who were directly approached, agreed to participate and were interviewed (74% recruitment rate). We are unsure how many people saw the study invitation through mailing lists for NHS email networks and professional bodies and as a result the overall response rate cannot be calculated. Interviews lasted between 34 and 92 min (median = 51 min).

Participant characteristics are summarised in Table 1. Most participants were between 45 and 54 years old, were at consultant level and had been in their current role for over one year. In total, participants from 14 different NHS trusts participated, with most based in the North Thames GMSA. Most (*n* = 16) participants were community-based at the time of interview; however, one was currently a hospital-based paediatrician, but whose recent training comprised 1 year in community paediatrics.

**Table 1:** Summary of participant characteristics

Participant Characteristics	Total *n* (%)
Age (Median, Range)	(50, 28–61)
25–34	1 (6%)
35–44	4 (23%)
45–54	9 (53%)
55–64	3 (18%)
Gender	
Male	5 (29%)
Female	12 (71%)
Grade	
Paediatrics Trainee	1
Registrar in Community Paediatrics	1
Specialty Doctor in Community Paediatrics	2
Consultant in General Paediatrics	1
Consultant in Community Paediatrics	12
Years in current post	
< 1	2
1–10	7
11–20	5
21–30	3
Number of NHS trusts represented	14
Participants by GMSA location	
North East and Yorkshire	2
East	1
North Thames	11
Central and South	3
Self-rated genomics experience	
A lot	6
Some	11
None	0

### Findings

**Fig. 2:**
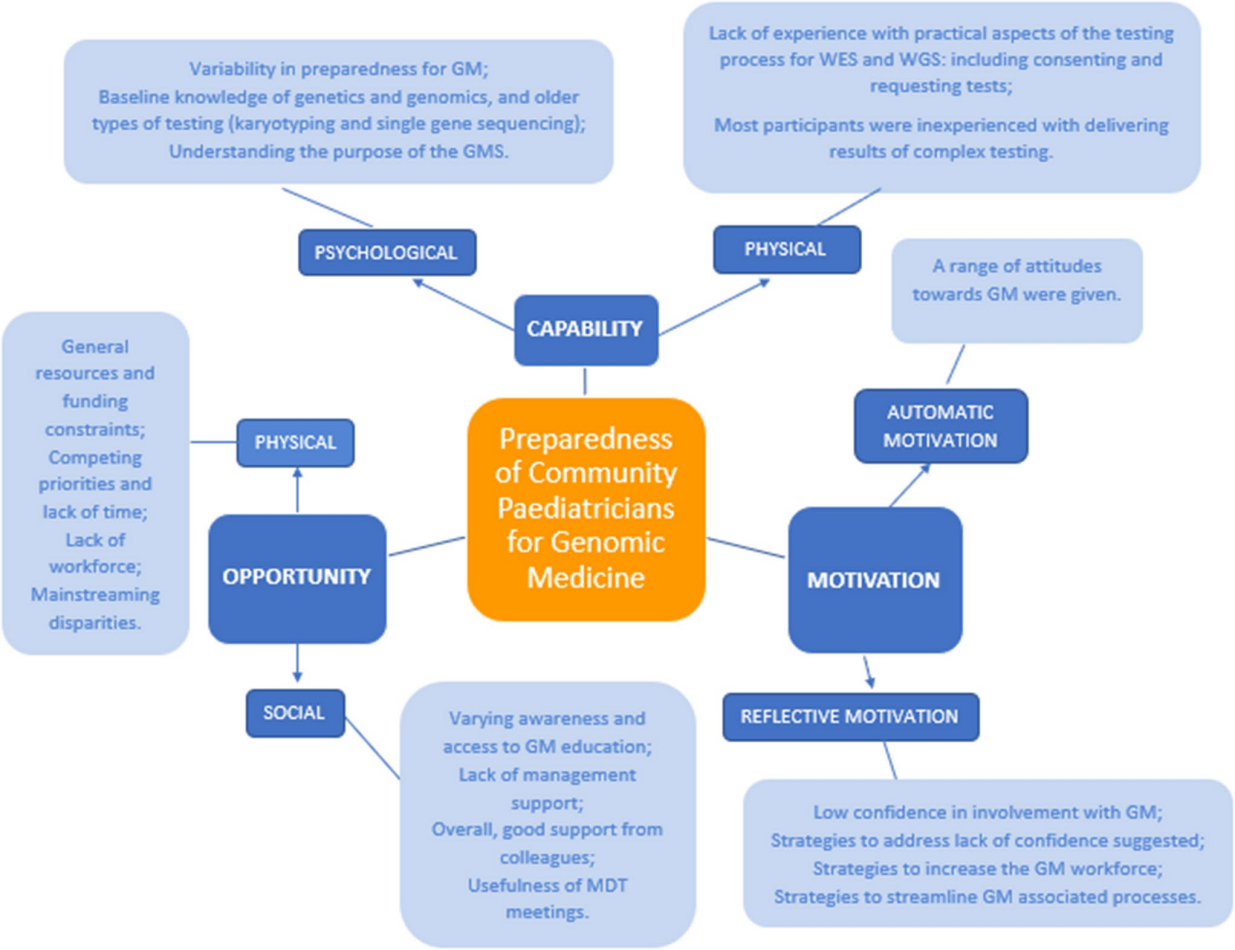
Radial cluster diagram summarising our study-specific findings within each sub-theme of the COM-B model

Our findings are presented according to the domains (in bold) and sub-domains (underlined) of the COM-B model: Capability (psychological and physical); Opportunity (physical and social); Motivation (reflective and automatic) along with the inductive codes within each of these subthemes (in italics). We have also highlighted where findings relate to strategies for interventions which most notably occurred in the Opportunity and Motivation domains. The study-specific findings are depicted diagrammatically in Fig. 2.

### Capability

The capability domain refers to whether participants have the knowledge, skills and abilities required to engage in a particular behaviour, in this context incorporating genomic medicine in their practice.

### Psychological capability

This relates to CPs’ preparedness for GM as well as their current perceived knowledge of genetics, genomics and the GMS.

#### Preparedness for GM

There was variability in how prepared participants felt they were to deliver GM in their practice. A lack of preparedness amongst participants was reported with some describing themselves as “not knowing enough”. One participant commented that:

*“I think if you asked me to write an essay on what I thought an exome and genome and what everything was*,* I think I’d fail.”* (Participant 13).

However, most understood that they would soon be required to have greater involvement with GM and therefore needed to upskill.

#### Knowledge of genetics and genomics

Most participants felt that they had a baseline understanding of genetic concepts, e.g., including inheritance patterns, monogenic conditions, and disorders seen more frequently in consanguineous populations.

*“So I was talking to them about. obvious syndromic and not obvious syndromic*,* and going through chromosomes*,* recessive and different syndromes. and mitochondrial diseases and things*,* so I could do that in my sleep actually.”* (Participant 13).

*“I’d say a lot of them* [patients] *do have consanguinity*,* so even when we don’t have a diagnosis from microarray or whatnot*,* you get the idea is that it probably is genetic in some degree”* (Participant 3).

Several participants made comments that highlighted their familiarity in identifying a child that might have a genetic condition and had ordered genetic tests in the past including microarray:

*“We can often see that there’s a family history or there’s a pattern and it doesn’t always show up anything on microarray but it’s quite clear that there’s something going on genetically”* (Participant 11).

Participants had a greater involvement with earlier forms of gene testing i.e., karyotyping, microarray and single gene testing, than advanced genomic testing. However, some participants demonstrated a greater understanding, referencing WGS diagnostic rates and the genes on specific panels. There was no observable pattern with regards to knowledge (about genomic testing) and participant characteristics (such as age or location). Some participants displayed an understanding of issues pertinent specifically to WGS, e.g., the greater chance of detecting variants of uncertain significance with WGS compared with when doing targeted tests:

*“If you already know exactly what monogenic condition you’re thinking about then it’s probably more appropriate just to test for that*,* considering we know that WGS has significant increased risk of variants of uncertain significance”* (Participant 3).

Overall, responses suggested participants were well-versed in the aspects of genetics that related to their specific roles and experience. Most participants had no formal genetics education prior to the initiation of the GMS and had acquired their knowledge through professional experience. Some reasoned that this was due to how long ago they trained in medicine; genomics was not part of their syllabus, and they trained before the development of advanced sequencing technologies.

#### Understanding the purpose of the genomic medicine service

All participants except one was familiar with the GMS. Participants identified several different objectives for the GMS; the most common related to improving patients’ access to genomic testing regardless of where in the country they live:

*“It was to make sure there was equitable coverage across England”* (Participant 13).

Some were familiar with the restructuring of regional services into the seven GLHs, with specific laboratories responsible for specialised testing: *“Experts level of analysis can take place in certain centres”* (Participant 3). Additionally, some participants referenced the National Genomic Test Directory, which was described as useful for standardising genomic testing for patients with a given phenotype regardless of geographical location.

### Physical capability

This relates to participants’ current level of ability to carry out GM-related work, for example, pre-test counselling, taking consent, ordering tests, interpreting and delivering results.

#### Experience with genomic testing: consenting and requesting tests

Most participants were experienced in consenting for earlier forms of gene testing, though not for larger panels, WES or WGS. Only a small number had consented patients for WGS in the 100,000 Genomes Project. There was an observable difference between the one hospital-based participant and community-based participants in terms of their experience consenting for WGS in the GMS. The hospital-based paediatrician had significantly greater experience: *“The Record of Discussion [consent form used for WGS] I’ve definitely done over say fifty times”* (Participant 16). In comparison, few of the other participants were well-versed with the consent process.

*“I think I’d have to do some homework in terms of the consent process”* (Participant 7).

One participant was critical about consent more broadly for genetic testing undertaken by non-genetics specialists:

*“The whole consent process that happens in the NHS*,* I mean as a genetic counsellor I think you would cringe when you hear how genetics are discussed in the majority of cases”* (Participant 12).

The minority of participants who had ordered larger gene panels, reported difficulties obtaining clarification on which genes could be tested individually vs. only on WGS testing.

#### Experience with genomic testing: delivering results

Participants acknowledged the complexities brought about by WES and WGS. Most participants had not yet delivered results from these tests but felt that they would be more confident in communicating results if they had direct experience with the condition:

*“If it’s straight-forward*,* if it’s a condition I know things about then I think I would feel comfortable. So*,* I think it just depends on the condition”* (Participant 12).

However, it was acknowledged that when microarrays were first introduced, CPs had to upskill in this technology and, in that respect, WGS was comparable.

### Opportunity

The opportunity domain refers to factors outside the control of the individual that impacts their adoption and implementation behaviour such as resources and time.

### Physical opportunity

This relates to the resources that are currently available for implementation of GM.

#### General resources and funding

Funding constraints underpinned many participants’ concerns with regards to implementing GM. Some described being asked to do something that they are “not funded for”, and healthcare being in “severe austerity”, which made the prospect of additional funding unlikely.

*“The thing is that we’re not trying to be difficult*,* it’s just that they keep giving us stuff without extra resources*,* time or money”* (Participant 15).

Relatedly, a further concern was balancing resources against the potential increase in children eligible for WGS. Participants reflected that it would be like “opening up a can of worms”. One participant estimated that they had around 250 existing patients who could be eligible for WGS.

*“What I’m not sure about is how ready genetics services are for quite how many patients there are going to be that would then be referred for genomic testing! … that’s almost every child that I see in my clinics!”* (Participant 3).

#### Time

Participants communicated being already overstretched, without the additional burden of organising genomic testing. They expressed concern that discussing the pros and cons of testing, consenting patients, and completing the associated forms would be excessively time-consuming. One described the burden of adding in these tasks to their existing workload as *“the straw that’s breaking the camel’s back”* (Participant 13).

Some participants felt that other areas of patient care would suffer if they had to include genomic testing within a standard appointment:

*“I just can’t see how I can do that without compromising on the rest of my clinical care for that patient”* (Participant 15).

#### Workforce

Around two thirds of participants commented on the lack of human resources to deliver GM within their roles. Participants highlighted a lack of clinical and administrative support for tasks including pre-test counselling, consenting, obtaining trio samples for WGS, and chasing blood samples. One participant summarised: *“I think the big elephant in the room that’s always there is ‘who’s going to do it?’”* (Participant 12). Others were more optimistic, suggesting that the process would become more streamlined over time, and would ultimately reduce resource burden.

Some participants perceived a workforce disparity between hospitals having seen support roles advertised at other sites.

*“What I think people have not understood very clearly is how certain regions have been able to get resources with genomic assistants*,* whereas other regions are saying no we can’t*,* we can’t provide that”* (Participant 1).

#### Involvement in mainstreaming

Participants agreed that offering genomic testing in the GMS was not a departmental priority and models of service delivery had not yet been established in any of their departments. There were disparities in participants’ experiences with regards to ordering WGS. In some, there was confusion about whether their specialty was permitted to order WGS:

*“It’s all been a bit confusing*,* so basically it’s like well I’m not doing that then*,* I’ll refer you to genetics!”* (Participant 13).

One participant knew of neurology colleagues within the same trust who were able to order WGS, an option not available to CPs. Only a small minority of participants reported already having ordered WES or WGS tests themselves.

### Social opportunity

This includes access to educational opportunities and support from colleagues, including genetics specialists and management.

### Education

Participants reflected on specific interventions such as education and training, that had been set up to upskill mainstream clinicians. Many participants had accessed educational sessions, delivered mainly by GMSA representatives and colleagues from Clinical Genetics departments. Participants had engaged with training opportunities on topics including the sequencing technologies available, paediatric mainstreaming, consenting and communication skills. The varying formats included virtual webinars, continuing professional development (CPD) modules, workshops and signposting to educational websites. Participants cited the online resources they had accessed genomics education through, including Genomics England, FutureLearn, the GMSA, local Clinical Networks, British Academy of Childhood Disability and the Royal College of Paediatricians and Child Health. A small number of participants had not accessed educational resources on GM, citing that they were not sure where to locate resources or whether the resources were intended for CPs. The following quote relates to accessing genomic medicine educational resources:

*“Where is it advertised? Where can you find out about these things? Because there must be people like me that are interested and just haven’t managed to find that education resource”* (Participant 6).

Most participants knew where to access GM educational resources but recognised a demand for more, and highlighted the need for improved accessibility amongst their colleagues:

*“It has happened quite quickly and yes there has been education*,* but not everybody’s been able to access it. I think a lot of people haven’t realised that they need to”* (Participant 4).

Competing priorities were mentioned as a barrier to participants accessing educational sessions.

*“This is the first time I’m attending tomorrow so it’s on a fixed day and then many people if they clinic on that day*,* we can’t attend”* (participant 2 referring to an educational genetics meeting).

*“But again*,* it comes down to time to be able to attend all these things and so forth. There isn’t unlimited time to access these things.”* (Participant 7).

The voluntary nature of the sessions also meant that engagement relied on the individual “drive” of CPs to engage with the educational resources. Notably, one participant had a colleague who *“didn’t believe in genetic testing*,* because he didn’t see the value of it”* (Participant 17) and this participant had worked to engage their colleague through resources evidencing its clinical utility.

#### Organisational structure and management support

Participants reported that there had been a lack of discussion and engagement initiated by those implementing the new service.

*“At a strategic level there did not seem to have been any conversations*,* which is really sad*,* and I would’ve expected that to happen”* (Participant 1).

Several participants expressed feeling left to *“*fight on their own*”.*

#### Support from colleagues

Overall, participants felt well-supported by their peers, including colleagues from clinical genetics. There were examples of colleagues from general paediatrics who were highly supportive in directing CPs to mainstreaming education. However, a small number of participants thought that their genetics colleagues should be taking on more of the work around genomic testing as it was their field of expertise and they perceived them to have more time allocated to this.

One interviewee suggested that the differences in engagement between their colleagues might be due to differences in the genomics training experienced by older and younger colleagues:

*“There’s that risk that often you’ll see that the trainees coming through and the younger members of the team are more au fait with this sort of stuff and the older stick-in-the-muds like me are kind of less well versed in it than some”* (Participant 7).

#### Multidisciplinary team (MDT) meetings and interest groups

Routinely organised MDT meetings were valued as being beneficial for up-skilling and seeking input for complex patients. Approximately half of participants had attended MDT meetings with clinical genetics colleagues. Many found the virtual nature of the meetings helpful, allowing them to join despite other clinical priorities.

### Motivation

This domain refers to the internal processes that influence decision-making in the context of carrying out the behaviour.

### Reflective motivation

This refers to participants’ confidence as well as their considered opinions about the GMS, including their desires to be involved with it, and suggested solutions and improvements to enabling GM.

#### Confidence in involvement with genomic testing

Several participants highlighted their hesitancy and discomfort in using WGS, mainly due to their unfamiliarity with the process and concerns about interpreting complex results.

*“People have anxieties about filling a form*,* simple as that. You know the first thing which comes to mind for a person who you are asking to – ‘which form do I fill?’ ‘Where do I send?’ ‘How do I do the test?’* (Participant 2).

Others described feeling less confident about their involvement, *“I’m sure we don’t do it as well as we should do and it’s very basic”* (Participant 14).

Many participants communicated that they would feel most comfortable referring a patient to clinical genetics for their support when more complex testing discussions were indicated. This included covering the “grey areas” of testing such as unexpected results and discussing additional complexities like non-paternity.

Strategies noted by participants to address lack of confidence amongst CPs included having greater involvement with GM, which participants expected would lead to an improvement in their competence and confidence over time: *“If they’ve [CPs] done it ten times they will feel more confident”* (Participant 2). They also appreciated the need to understand the “nuts and bolts” of the process, referencing practical elements such as navigating the lengthy National Genomic Test Directory, using the correct forms, and knowing the correct testing turnaround times.

#### Workforce

Other strategies identified by participants to counter the behavioural barriers to implementing GM in community paediatrics related to the workforce. This included additional funding to recruit staff such as genomic associates whose main roles would be to support the department, including: consenting patients, completing paperwork, chasing samples and submitting test requests to the lab. They felt that this would ensure the GM processes were “joined-up”. They recognised that employing someone to this role would be cheaper and more efficient than a consultant having to take on this work which was currently taking away valuable time from their main role. Participants also proposed developing a ‘genomics champion’ role within their community paediatrics team who would be responsible for promoting team training, and support colleagues with consenting patients and delivering results.

#### Processes

Suggestions were also made to streamline the WGS process included improving pathways, making consent procedures tighter, and reducing the paper-based workload. Some participants proposed more easily accessible guidelines, “cheat-sheets” and provision of standard operating procedures, for example, around how many times to chase missing consent forms or blood samples.

### Automatic motivation

This refers to the more instinctual and impulsive processes and opinions about the GMS held by participants, for example, whether they perceive it to be valuable.

#### Attitudes towards GM

Many participants had a positive attitude towards GM, viewing it as having an important place within CP: *“The value added I have no question about it*,* it is adding to our understanding”* (Participant 18). Other participants foresaw their roles evolving to have a specialist interest in genomics and felt that genomic tests should be standard practice and *“become [an] inseparable part of our job*” (Participant 5).

The potential benefits for patients were cited as being key drivers behind their motivation for supporting the implementation of GM. They thought that the diagnostic potential in using WGS offered great opportunity for families without diagnoses.

Most participants were receptive to the implementation of GM and thought that their colleagues would feel similarly. Some described their own excitement at the prospect of utilising advanced genomic testing and described their colleagues as passionate. However, a small number of participants perceived a lack of receptivity from their colleagues, particularly those who were more senior and closer to retirement who were perhaps less open to learning something new.

*“There was some apprehension from more senior members of the team who are maybe closer to retirement stage and how they then learn something new and different”* (Participant 11).

## Discussion

The introduction of a disruptive technology into clinical practice is bound to be accompanied by challenges. In this study, we asked CPs how prepared and confident they felt regarding the implementation of GM into routine care in the NHS in England. Our findings, informed through the lens of the COM-B model, have highlighted that barriers to behaviour change exist across the domains of capability, motivation and opportunity. There is wide variation across trusts in CP preparedness for genomic medicine for reasons including lack of time and resources, notably workforce support. Many also lack confidence in the skills required to deliver GM. Most participants perceived a net benefit from GM for their specialism, were receptive to change, and foresaw its potential for improving both clinical management for their patients, the provision of information for families, and felt that their clinical competence would improve with repeated exposure. However, there was a prevailing viewpoint that GM was not currently a top priority. Participants reflected that they were working in an already overstretched healthcare system, with many competing priorities, and some responses suggested that adding genomic testing to the workload of CPs was not feasible.

Participants identified a number of strategies for interventions that either already or potentially could support adoption of GM by CPs, many of which align to the intervention functions and/or policy categories of the COM-B Behaviour Change Wheel (Michie et al. 2011). These include ‘fiscal measures’ e.g. funding for additional workforce such as genomic associates, and development of ‘genomic champion’ roles, aspects related to ‘enablement’ such as streamlining of administrative aspects and development of standard operating procedures, ‘training’ through greater involvement with GM colleagues, and ‘education’ notably improved accessibility to educational opportunities. While there are numerous educational initiatives aimed at increasing genomic literacy, our findings suggest that these are not always well-aligned with the needs of CPs. Most participants felt that they had access to GM education, but the main challenge was their capacity to engage with it. Whilst the centralised model of the GMS aims to address fair distribution of resources, it appears from our findings that implementation has progressed at different rates across the country, leading to inequity in its delivery.

Our findings are consistent with a number of other studies looking at preparedness for genomic medicine amongst mainstream clinicians across the UK and more globally. Our finding that most participants felt that GM would benefit their patients are consistent with published studies that have surveyed a variety of non-genetics HCPs who agreed that GM will improve diagnosis and health outcomes for patients (Park et al. 2021; Rosso et al. 2020; Carroll et al. 2016; Crellin et al. 2019; Peter et al. 2024). However, the finding that many did not see GM as a priority, has been found elsewhere in the literature, for example, a study conducted in the UK with general practitioners agreed with some participants for whom genomics simply was not a priority anywhere near the top of their workloads (Mwale and Farsides 2021). In an Australian study with nephrologists, participants cited inadequate staffing and lack of funding as key barriers to implementation (Jayasinghe et al. 2020).

We also found in our study that whilst participants had a baseline understanding of genetics, understanding of genomics was patchier and some felt they did not know enough to competently consent and return results to their patients. Other studies conducted in the UK, Canada and Europe with primary care physicians, gastroenterologists and public health professionals have also reported participants’ self-rated genomics competence as poor or lacking (Carroll et al. 2019; Rosso et al. 2020; Bakir et al. 2019).

Most participants felt they lacked the capacity to engage with educational initiatives aimed at upskilling them in genomics because of having too many other, more pressing, clinical priorities, a finding which has also been echoed in other studies (McClaren et al. 2020b; Peter et al. 2024). Notably, however, we did recognise a demand for learning about GM to improve confidence in their involvement with WGS. Educational programmes in England, such as the Genomics Education Programme, have been designed to offer HCPs tailored information about GM to consolidate their knowledge (Genomics Education Programme 2022). The GMSAs also play a role in embedding GM into mainstream healthcare within their assigned geographical region (Hill 2020), and are organising training and education for non-genetics HCPs in the form of webinars and training sessions, striving to be ‘easily accessible’ for busy HCPs (South East Genomics 2022). Virtual drop-in sessions and ‘lunch-and-learn’ hours, have also been designed since the initiation of the GMS, to provide non-genetics HCPs access to digestible educational materials, accessible resources, and provide a forum for answering questions. Flexible and accessible learning options such as GeNotes (NHS England 2024a; NHS England 2024b) have more recently been developed, and offer quick, concise information to help healthcare professionals make genomic testing decisions at each stage of the clinical pathway. These aim to make the information accessible to clinicians at their discretion, and provide an alternative for clinicians to access it they cannot attend a virtual session, due to other priorities. Easily accessible, nationally standardised decision-aids for real-time support in the clinic for non-genetics HCPs have been recommended in the literature (Carroll et al. 2019; Salm et al. 2013).

In terms of embedding genomics within medical training, an advanced subspecialty core module in genomics for all CP trainees could promote competence and therefore confidence in knowledge around GM. A study involving UK-based non-genetics medical trainees identified that they supported incorporation of GM modules into their specialty curriculum (Bakir et al. 2019). This has already been successfully incorporated for cardiology specialists by the Joint Royal Colleges of Physicians Training Board, for an advanced subspecialty module in inherited cardiovascular conditions (Joint Royal Colleges of Physicians Training Board 2023). Advancing genomics education within mainstreaming teams has already had some success, with some non-genetics HCPs taking on the role of becoming knowledgeable about GM and mainstreaming in their department (a role known as ‘genomic champions’). With the advent of GMSAs there is greater potential for creating more educator roles within teams to disseminate training amongst colleagues (Slade and Burton 2016). Such roles could help CPs feel reassured by having support from a colleague within their department who could help them with genomic testing queries. Employing genomic associates to take consent and help facilitate the consent process could also potentially free up CPs to focus on their routine clinical work as well as return WGS results.

Our findings also reflected that some participants had identified disparities, from their own experiences, in a service where one of its key aims was reported as providing equitable care across the country (NHS England 2020), whilst their own experiences disputed this. The disparities included differences in the workforce resource commissioned to assist with WGS in community paediatrics, and that some of their non-CP colleagues were able to order WGS for conditions they would also see, whereas they were not. Moreover, there appeared to be geographical differences between the level of GMSA support that participants received. Standardised practices and processes are important across trusts to incentivise engagement with GM.

Leadership engagement is important when a new intervention is adopted as it has been shown to promote involvement of the wider team (CFIR Research Team 2021; Slade and Burton 2016). When an intervention is perceived as useful to one’s clinical practice, engagement will likely increase and teams will become more invested in the new service (McClaren et al. 2020a). This is consistent with the rationale behind the GMSAs which were established to systematically facilitate the implementation of GM into the wider NHS (Lewis-Beck et al. 2011). Demonstrating the downstream benefits of GM to mainstream clinicians including CPs may increase willingness to adopt the new system, as might ensuring the process of engagement is as simple. This might include, for example, more streamlined consent processes, a centralised electronic system, and clear ‘decision-trees’ and referral pathways for when it is appropriate to refer a patient on to clinical genetics. Regular feedback between CPs and those responsible for the implementation of the GMS would also ensure any issues could be ironed out as early as possible.

### Limitations

In this study, participants from a range of geographical locations and with differing levels of experience within CP were interviewed. However, only a small number of trusts across England were represented and covering only four of the seven GMSAs, which may limit the generalisability of the findings across the country. Ascertainment bias may have impacted the data, as those with the strongest opinions and highest interest in the GMS may have responded to the invitations for participation compared with those who did not. We also relied on a snowball sampling approach, where known prospective participants were directly contacted by the study as we were unable to recruit participants through mailing lists. Additionally, there were a higher number of female participants than males (71% compared with 29%) and older participants were over-represented. However, as reported in the workforce census, the CP workforce is majority female and older, therefore the study participants may actually be more representative of the real workforce composition (Royal College of Paediatrics and Child Health 2022).

## Conclusion

CPs are a key group of clinical specialists likely to benefit from adopting GM in their practice, but in order that they feel prepared and confident to engage effectively, overcoming the early obstacles that we have identified through this study will require investment, resources and support in order to influence behaviour change. Further research with CPs in the future would be useful to explore whether confidence and preparedness for GM has improved and to refine strategies to support implementation. Further analysis of the findings using determinant frameworks such as the Theoretical Domains Framework as well as the CFIR could provide a more nuanced understanding and serve to pinpoint more specific barriers and facilitators (Cane et al. 2012). This could in turn help to more precisely identify the behavioural determinants of preparedness and tailor interventions to effectively address these gaps. Finally, our research provides one of the first accounts of mainstream clinicians grappling with the new genomic landscape. Our findings may be relevant to clinicians in other non-genetic specialties integrating genomic medicine into their clinical practice not only in the UK NHS but more globally.

## Electronic supplementary material

Below is the link to the electronic supplementary material.

Supplementary Material 1

## Data Availability

The data that support the findings of this study are available from the corresponding author upon reasonable request and where participant consent has been given.
