# Supplementary material for: A qualitative study to evaluate the preparedness of community paediatricians for genomic medicine in England - ready for take-off?
Source: J Community Genet. 2025 Mar 12;16(3):321–34. doi: 10.1007/s12687-025-00781-8 (PMC12202262; doi:10.1007/s12687-025-00781-8)
Supplement: Supplementary file 1 — Supplementary Material 1 [file 12687_2025_781_MOESM1_ESM.docx]

**Supplementary Information**

**Journal of Community Genetics**

**Study title: A qualitative study to evaluate the preparedness of community paediatricians for genomic medicine in England. Ready for take-off?**

**Running title:** Community paediatricians’ preparedness for genomic medicine

Sophie Marlowe^1^, Melissa Hill^2,3^, Michelle Peter^2,3^, Celine Lewis^2,4^

^1^ North East Thames Clinical Genetics Service, Great Ormond Street Hospital, London, United Kingdom

^2^North Thames Genomic Laboratory Hub, Great Ormond Street Hospital for Children NHS Foundation Trust, London, UK.

^3^Genetics and Genomic Medicine, UCL Great Ormond Street Institute of Child Health, London, UK.

^4^Population, Policy and Practice Department, UCL Great Ormond Street Institute of Child Health, London, UK

Corresponding author: Sophie Marlowe, [Clinical Genetics Unit, Great Ormond Street Hospital, Great Ormond Street, London WC1N 3JH](mailto:gos-tr.clinicalgenetics@nhs.net)

Email: [sophie.marlowe@nhs.net](mailto:sophie.marlowe@nhs.net)

**Interview Topic Guide**

INTRO:

Thank you very much for agreeing to take part in this one-off interview about the new Genomic Medicine Service.

The interview is semi-structured and you can answer questions as briefly or detailed as you are able to.

- Please could you confirm whether you have read the information sheet?
- Do you have any questions at this stage?
- I will now read out the consent form to take your verbal consent for participation.

[READ CONSENT FORM]

I will now ask you a few starter questions before we begin.

Professional Background questions

1. Age:
2. Gender:
3. What is your clinical specialty?
4. What is your current job title?
5. How long you have been in your current role?
6. Which hospital do you work in?
7. Which department do you work in?
8. Patient type seen:
   1. Adult patients
   2. Paediatric
   3. Both
9. Genomics experience (discussing genomic tests with patients):
   1. None
   2. Some experience
   3. A lot of experience
10. Did you consent any patients to the 100,000 Genomes Project?

[Prompts: How many years have you worked as an “…….” / which organization do you work at? / how long have you been working there?]

I **am going to start by asking you some questions about the Genomic Medicine Service.**

1. What was the rationale of the development of the GMS [prompt: what does it build upon; why is the time right for implementing genomic medicine into clinical practice; aid with diagnosis for children]
2. What do you think the overall aims and intentions are of the GMS?
   1. Short term aims [prompt: improve diagnostic yield, reduce diagnostic odyssey, equity of access, mainstreaming genomics]

Longer term aim [prompt: genomic industry, research, data sharing, contribute genomic data to databases]]

1. What needs to happen to achieve these aims?
2. Before I ask you about you anything further, I would be interested to hear (broadly speaking) about your overall opinion of the GMS so far.

**I am now going to ask you about your experience of genomic testing**

1. How do you feel about the implementation of genomic testing – so what I mean by that is gene panels, WES/WGS) into your routine practice?
   1. To what extent do you think that the change brought by genomic testing is needed within your specialty? [i.e. mainstreaming genetic testing, streamlining processes, standardizing testing)]
2. Can you tell me about your experience of offering genetic or genomic tests before the newly commissioned GMS?
   1. What tests were you ordering? [prompt: microarrays, single gene tests)
   2. Were you consenting patients?
   3. Returning results?
   4. Attending MDT meetings with the genetics team?
3. Can you tell me what’s changed in terms since the newly commissioned GMS?
4. Are there new tests that you are ordering that you weren’t ordering before?
5. If yes, what tests are they? (prompt, gene panels, WES/WGS)
6. How is this different to what was happening before?
7. Have you offered WGS to any of your patients?
8. If NO –why? [Prompt: you haven’t been able to request WGS for your patients? don’t have suitable patients?]
9. IF YES: Can you give me any examples? [Prompt: for which indications/conditions?]
10. IF YES: Approximately how many patients/parents have you discussed WGS with?
11. IF YES: What has been your experience of consenting patients for WGS? (prompt: consent discussion/completing record of discussion form/test ordering)
12. If YES: Have you returned any WGS results to patients? What has been your experience?
13. What are your thoughts on using WGS for rare disease diagnosis?
14. Do you think it is appropriate/ a good test to offer to patients?
15. What do you think the benefits are of WGS? [Prompt: more benefits for children with complex conditions for example complex epilepsy and intellectual disability compared with traditional management? Cost-benefit/economics]
16. Do you have any concerns about WGS?
17. What have been some of the challenges that you yourself are currently facing in terms of offering WGS? [prompt: time taken to consent patients; understanding/confidence with returning results report]
18. What support has been put in place to support you in providing WGS to your patients?
19. Has any additional support been put in place e.g. new roles such as genomic practitioners, whose role it is to assist with taking consent, sample collection and tracking, delivery of results etc? [Prompt: also, genetic counsellor involvement?]
20. What, if anything, has worked particularly well in terms of support you have been given? [Prompt: has there been any support from those above e.g. local genetics teams, the GMSA, clinical research networks?]

**I’m going to ask you some questions now about how prepared you feel for genomic medicine**

1. How **prepared** do you feel to offer genomic medicine including WGS in your clinical practice?
2. How would you describe your own **knowledge and understanding** of genomic testing including WGS?
3. Have you accessed any genomics education? If yes, what type of educational material (i.e. formal course, attended training delivered, independent study)?
4. [If yes to above] Have you delivered any educational sessions or materials for colleagues?
5. [If no to above] Do you know where to go to access genomics education (i.e. GEP/HEE, GMSA or local resources such as training sessions by genetics teams)?
6. How **confident** do you feel about your abilities to deliver genomic medicine including WGS?
7. How confident do you feel in **identifying suitable patients**?

(prompt: Do you know where to find information on eligibility criteria for genomic testing i.e. the genomic test directory? have you used this before?

1. How confident do you feel to **consent** patients?
2. How confident do you feel to **return** results?

**Now I am going to ask you some questions about how prepared your department is for genomic medicine.**

1. How well is your department set-up for genomic medicine including providing WGS (i.e. how receptive are your colleagues to this change, education & training, how well supported are they by senior team members, i.e. sharing knowledge, and are there any key implementation targets to be met?)
2. How has your department responded and adapted to genomic medicine?
3. What are some of the challenges that the department as a whole are facing with regards to implementation of genomic medicine? [prompt: the testing pathway (logistics), communication, lack of training, staffing, bioinformatics/data, infrastructure]
4. Are there sufficient resources in your organization for the implementation of genomic medicine? (i.e. staffing, funding and investment in training)
5. Can you tell me about any changes that have been made in your department in order to integrate genomic medicine into your everyday practice? (i.e. using the new genomic test directory and different genetic tests, organizing more genetic testing than before, doing more pre-test genetic counselling)
6. How confident do you think your colleagues feel about offering genomic medicine including WGS in their practice?
7. What else do you think could be done to improve the implementation of genomic medicine within your department? (i.e. leadership engagement, more resources available, better training and education)
8. What has been your experience in terms of support available from those designing and implementing the GMS for the introduction of genomic medicine e.g. NHSE, GLH, GMSA leads, CRNs? Have you had any interaction with them?

Is there anything else you would like to share with me that I have not asked you about?
